# Adaptive responses to elevated CO_2_ in fruit species with different phloem loading mechanisms

**DOI:** 10.3389/fpls.2024.1356272

**Published:** 2024-08-01

**Authors:** Marzieh Davoudi, Spyridon Kalantzis, Antonios Petridis

**Affiliations:** Department of Food Science, Aarhus University, Aarhus, Denmark

**Keywords:** apoplastic loader, carbon dioxide, carbon allocation, fruit crops, phloem loading mechanism, photosynthesis, symplastic loader

## Abstract

**Introduction:**

It has been suggested that the mechanism of phloem loading, that is apoplastic or symplastic loading, may affect a plant’s ability to adapt to elevated CO_2_ levels. Strawberry (*Fragaria × ananassa*) and tomato (*Solanum lycopersicum*) are two fruit crops that use different mechanisms to load sugars into the phloem – the former symplastically and the latter apoplastically – yet both species can increase their yields when grown in a CO_2_-enriched environment. In this study, we subjected strawberry and tomato plants to long-term CO_2_ enrichment to determine the morphological and physiological adaptations that enable them to increase their yields in response to higher CO_2_ levels.

**Methods:**

Transplanted tomato and strawberry plants were subjected to ambient (400 ppm) and elevated (800 ppm) CO_2_ for three months. We examined various parameters associated with growth, yield, photosynthesis, and carbon allocation by means of phenotyping, gas exchange analysis, and ^13^C labelling combined with isotope ratio mass spectrometry.

**Results:**

We found that CO_2_ enrichment promoted growth and reproductive development in both species, resulting in more flowers per plant (tomato and strawberry), larger crown (strawberry), and, eventually, higher yields. Gas exchange analysis and *A*/*c*_i_ curves revealed that elevated CO_2_ increased carbon assimilation rate in strawberry, but not in tomato – the latter being limited by Rubisco’s carboxylation efficiency. Finally, whereas both species prioritized fruit development over the development of other sink organs, they were both limited by carbon export at elevated CO_2_, since new photoassimilates were equally distributed to various sinks between CO_2_ treatments.

**Discussion:**

The findings suggest that both species will benefit from future increases in CO_2_ levels and support current glasshouse practices entailing CO_2_ enrichment. Those benefits probably stem from an enhanced performance of both species at early developmental stages, as differences in carbon assimilation rate (tomato) and carbon allocation between treatments at late developmental stages were absent. Moreover, crop adaptation to elevated CO_2_ seems to depend on the ability of each species to respond to elevated CO_2_, rather than on the phloem loading mechanism per se.

## 1 Introduction

Phloem loading is the first step in photoassimilate translocation from source leaves to heterotrophic sink organs (e.g., roots, flowers, and fruits), comprising the transport of photoassimilates from photosynthetic mesophyll cells to the long-distance transport tissue, the phloem (Rennie and Turgeon, 2009; Ainsworth and Lemonnier, 2018). Depending on the species, plants use primarily two distinct routes to load sugars (mainly sucrose) into the phloem, involving either an apoplastic or a symplastic pathway. In apoplastic loading, sucrose moves from mesophyll cells to the cell wall space (apoplast) and is subsequently loaded energetically into the phloem by the action of specific transport proteins, including members of the SWEET and sucrose transporter (SUCs or SUTs) protein families. In symplastic loading, sucrose moves cell-to-cell towards phloem through numerous narrow cytoplasmic channels, called plasmodesmata (Rennie and Turgeon, 2009; Braun et al., 2014; Ainsworth and Lemonnier, 2018).

Current evidence suggests that several features associated with phloem loading and carbon export are subject to environmental control to balance source supply with sink demand (Amiard et al., 2005; Adams et al., 2007; Bishop et al., 2018; Xu et al., 2018). These responses to environmental cues involve anatomical and molecular changes and depend on the mode of phloem loading. For example, growth under high light conditions resulted in a higher number of cell wall invaginations in minor vein companion cells to facilitate more sugar transporters (apoplastic loaders), or in a higher vein density to increase total plasmodesmatal frequency (symplastic loaders), ensuring in both cases greater delivery of photoassimilates to heterotrophic organs as a result of higher photosynthetic rates (Adams et al., 2007). Similarly, environmental stresses that limit carbon assimilation rate reduced SUTs expression in various apoplastic species (Xu et al., 2018).

A current gap in our knowledge is how plants with different phloem loading mechanisms respond to elevated CO_2_ with only few studies having dealt with this question so far (Körner et al., 1995; Bishop et al., 2018). Körner et al. (1995) investigated the accumulation of non-structural carbohydrates in leaves of apoplastic and symplastic species to test the hypothesis that symplastic species are less efficient than apoplastic species in exporting carbohydrates into the phloem for long distance transport to heterotrophic organs. The authors additionally hypothesized that if symplastic species were limited in their ability to export carbohydrates into the phloem, then they would have exhibited an excess of non-structural carbohydrates in their leaves when subjected to elevated CO_2_ conditions (Körner et al., 1995). Indeed, the authors found a higher accumulation of non-structural carbohydrates in the leaves of most symplastic species, but they did not investigate further what would be the impact of this differential accumulation of non-structural carbohydrates between symplastic and apoplastic species on their photosynthesis or other important agronomic parameters such as yield. Likewise, Bishop et al. (2018) examined the impact of elevated CO_2_ levels on photosynthesis and carbohydrate accumulation in leaves of three apoplastic (pea, beet, and sugar beet) and three symplastic species (strawberry, melon and peony). The authors concluded that species differing in phloem loading mechanism had similar photosynthetic responses to elevated CO_2_, while, contrarily to Körner et al. (1995), they did not observe a higher sucrose build up in the leaves of symplastic species compared to apoplastic species. Again, however, there was no information about the distribution of photoassimilates to different sink organs or changes in yield in response to elevated CO_2_.

Strawberry (*Fragaria × ananassa*) and tomato (*Solanum lycopersicum*) are two important fruit crops that use different mechanisms to load sugars into the phloem – strawberry is a symplastic loader (Rennie and Turgeon, 2009; Bishop et al., 2018) and tomato is an apoplastic loader (Osorio et al., 2014) – yet both species benefit from CO_2_ enrichment by increasing their yields (Mamatha et al., 2014; Tagawa et al., 2022). In this study, we investigated the long-term effects of CO_2_ enrichment on strawberry and tomato plants to determine the morphological and physiological adaptations that enable these two contrasting crops to adapt and benefit from elevated CO_2_ levels. Determining how these two species respond to elevated CO_2_ levels could eventually inform practices involving CO_2_-enrichment in the glasshouse and reveal potential barriers that may limit their productivity.

## 2 Materials and methods

### 2.1 Plant material and growth conditions

The experiment took place at the glasshouse facilities of the Department of Food Science, Aarhus University, from November 2022 to March 2023. Seeds of tomato (*Solanum lycopersicum*) cultivar ‘Roma’ were obtained from a commercial supplier (SeedCom A/S, Denmark), sown directly in peat substrate in 96-cell plastic trays, and grown to ~ 4-week-old seedling stage according to standard protocols. The everbearing strawberry (*Fragaria × ananassa*) cultivar ‘Bravura’ was propagated from our own stock (initial plants were obtained by SW Horto A/S, Denmark) by cutting runner tips from mother plants and planting them in 50-cell plastic trays to develop roots.

In mid-November 2022, newly established tomato and strawberry plants were transplanted in 5.5 L plastic pots containing commercial peat substrate (Pindstrup 2; Pindstrup Mosebrug A/S, Ryomgaard, Denmark) and transferred to two separate but adjacent glasshouse rooms, corresponding to each of the two CO_2_ treatments. For each fruit species and each treatment, we placed 13 plants in the glasshouse rooms that were selected based on their uniformity and vigor. In the control treatment, CO_2_ was applied at 400 ppm concentration, while in the elevated CO_2_ treatment, CO_2_ was applied at 800 ppm concentration, the latter corresponding to projected CO_2_ levels for the year 2100 (Valone, 2021). Both species were grown under long-day conditions (16 h light and 23-25°C day-temperature/8 h dark and 18-20°C night temperature) with natural and supplemental artificial light (~320 to ~ 720 μmol m^-2^ s^-1^), the latter provided via LED lamps (FL300 Grow, Senmatic A/S, Søndersø, Denmark). The relative humidity in the glasshouse was maintained between 50-60%. All plants were fertigated with fertilizer suitable for tomatoes and strawberries by flooding the tabletop for 12 minutes twice a day. To ensure pollination of strawberry and tomato plants, flowers of both species were brushed at the full bloom stage using a paint brush. Since plants were flowering throughout the experimental period, we ensured that plants were brushed two to three times per week until the end of the experiment. Any damaged or diseased plants were excluded from further analysis. Finally, tomato plants were only lightly pruned to reduce the probability for disease infections but at the same time resemble how they are being produced in the field for industrial purposes.

### 2.2 Plant phenotypic analysis

Phenotypic analysis was performed to confirm the beneficial effect of elevated CO_2_ on growth and productivity of both species, and to determine which phenotypic characteristics may have contributed to any of those benefits. For most traits and both species, phenotypic analysis was performed at weekly intervals. This time-course evaluation of certain phenotypic traits was necessary in order to determine how they change in time in response to CO_2_ enrichment, which in turn would indicate the timing in which potential benefits may occur for both crops. Besides a basic understanding of when the two crops can capitalize on CO_2_ enrichment, this knowledge is also important because it can inform management practices in the glasshouse (e.g., to inform the duration of CO_2_ enrichment period).

For tomatoes, we measured primary shoot length (length between first leaf and apical meristem), number of inflorescences per plant, number of open flowers, closed flowers and fruits, flowering time, and yield. For strawberries, we measured leaf length and width, crown diameter, flower number, and yield.

Regarding tomato phenotypic traits, primary shoot length was measured with standard 30 cm or 100 cm rulers, while the number of inflorescences and flowers/fruits per inflorescence were measured by counting. Fruit yield was measured by weighing all fruits of a plant when 75% of the fruits had turned red.

For strawberry phenotypic traits, leaf length and width were measured using a ruler, while crown diameter using a digital caliper (Fowler, Cole Parmer, UK). Yield was measured by weighing all berries of a plant when the most mature fruits of the plant were on the ‘brick red’ stage.

### 2.3 Photosynthetic gas exchange measurements

Gas exchange measurements were conducted at the fruiting stage on the youngest, fully developed leaf of tomato and strawberry plants according to Petridis et al. (2018) with minor modifications related to temperature and gas flow rate. For each species and treatment, we used three independent replicate plants. Net carbon assimilation rate (*A*), stomatal conductance (*g*_s_), and intercellular CO_2_ concentration (*c*_i_) were measured using a GFS-3000 portable gas exchange system (Heinz Walz GmbH, Effeltrich, Germany) equipped with a 2.5 cm^2^ leaf cuvette, which provided light through an integrated LED light unit. Leaf temperature was maintained at 23˚C and relative humidity at 60%. Except for *A*/*c*_i_ curves, CO_2_ concentration was supplied at 400 (for control) or 800 (for elevated CO_2_) µmol mol^-1^ with a gas flow rate of 300 mL min^-1^.

*A*/*c*_i_ curves were generated at 1200 µmol m^-2^ s^-1^ using the following stepwise gradients: 400, 200, 100, 50, 400, 500, 600, 800, 1000, 1200, 1400, and 1600 µmol mol^-1^ (Petridis et al., 2018).

### 2.4 Labeling with ¹³C isotope

Labeling with ^13^C isotope was performed in both tomato and strawberry plants. In total, we used five tomato (three replicates for eCO_2_ and two replicates for control treatments) and five strawberry plants (three replicates for eCO_2_ and two replicates for control treatments). Whole plants were enclosed in transparent plastic bags, transferred in a growth chamber, and labelled for 2 hours. To generate ^13^CO_2_, 3 ml of 70% lactic acid was injected into glass vials, containing 1 g of NaH^13^CO_3_ (^13^CO_2_, treated plants) or NaH^12^CO_3_ (^12^CO_2_, control plants). The glass vials were mounted on the pots before covering the plants with the bags. When the labelling period ended, the bags were removed. ^13^C was chased for 24 h before harvesting the plants.

After chasing for 24 h, the above ground organs (fully developed and developing leaves, flowers, fruits, and crown) were separated and immediately frozen in liquid N_2_. The roots were washed to remove soil and then frozen to liquid N_2_ too. Frozen samples were placed in an oven at 70°C for 3 days, milled to fine powder, and stored at room temperature until elemental analysis.

The stable carbon isotopic composition (δ ^13^C) and carbon content of lyophilized powdered material were analyzed by OEA Laboratories LTD (Callington, UK) using a dual-pumped Sercon 20-20 isotope ratio mass spectrometer (IRMS, Sercon Ltd, Crewe, UK) coupled to a Thermo EA110 elemental analyzer (Thermo Fisher Scientific, Waltham, MA, USA). For each sample, approximately 0.94-1.2 mg of tissue were weighed, and 2 standards were used to calibrate the data (USGS L-glutamic acid and USGS41a L-glutamic acid). Excess δ ^13^C (%) of a given organ was calculated by subtracting δ ^13^C values after 24 hours of chasing from δ ^13^C values of control plants.

### 2.5 Data analysis

The exact number of individuals (*n*) used for quantitative analysis are presented in each figure. Depending on the examined parameter, we used a minimum of 3 and a maximum of 13 replicates, with the only exception of the control treatment of ^13^C labeling experiment in which we used 2 replicates. In the latter case the analysis is valid, but the statistical power is lower. Data analysis and calculation of 95% confidence interval of the CO_2_ response curves were performed using GraphPad Prism, version 9.5.1 (Dotmatics, Boston MA, USA). Data from ^13^C labeling experiment were subjected to two-way analysis of variance (ANOVA). Significant differences (*P ≤* 0.05) between means were determined using either Tukey’s test or unpaired t-test.

## 3 Results

### 3.1 Phenotypic responses to elevated CO_2_

To determine the effect of elevated CO_2_ on tomato and strawberry plants, we assessed several parameters associated with vegetative growth and productivity, across the entire experimental period. Specifically, for tomato we measured plant height, number of inflorescences per plant, number of flowers and fruits per plant, and yield, whereas for strawberry leaf length and width, crown diameter, number of flowers and fruits per plant, and yield.

**Figure 1:**
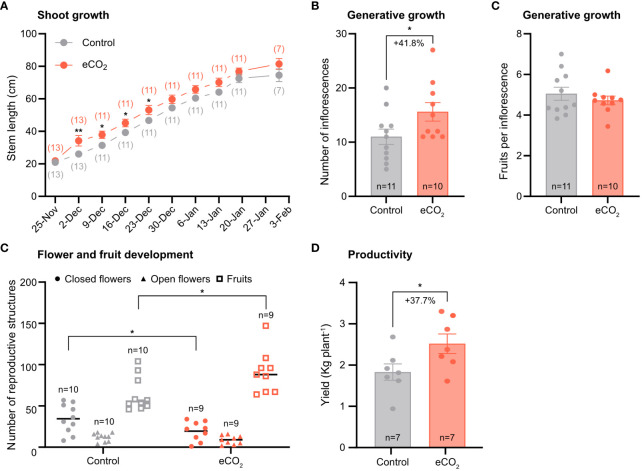
The effect of elevated CO_2_ on growth and yield-related parameters of tomato plants. **(A)** Stem length. The numbers in parentheses indicate the number of plants used in the analysis. **(B)** Number of inflorescences per plant and fruits per truss. n, number of plants used in the analysis. **(C)** Number of fruits per inflorescence **(D)** Total number of reproductive structures. **(E)** Yield. Error bars indicate the mean standard error, while dots in each bar represent the number of replicates. Percentage indicates changes in number of inflorescences and yield in response to CO_2_ enrichment. Significant differences (P ≤ 0.05) between means were determined using t-test. The asterisks above data points indicate significant differences (*P ≤ 0.05, **P ≤ 0.01).

Overall, tomato plants grown under elevated CO_2_ levels had a higher growth rate compared with plants grown under ambient CO_2_ levels, specifically during the first month of the experiment, corresponding to the vegetative growth stage (**Figure 1A**).

Regarding the generative structures, tomato plants grown at elevated CO_2_ had more inflorescences per plant compared with plants grown at ambient CO_2_ (**Figure 1B**). However, the number of fruits per inflorescence was similar between treatments (**Figure 1C**). In addition, CO_2_ enrichment accelerated transition to fruiting, as evidenced by the lower number of closed flowers and the higher number of fruits measured in the elevated CO_2_ treatment compared with the control treatment. Finally, CO_2_ enrichment resulted in a 37.7% yield improvement (**Figure 1E**), presumably as a result of the greater number of inflorescences measured in that treatment (**Figure 1B**).

**Figure 2:**
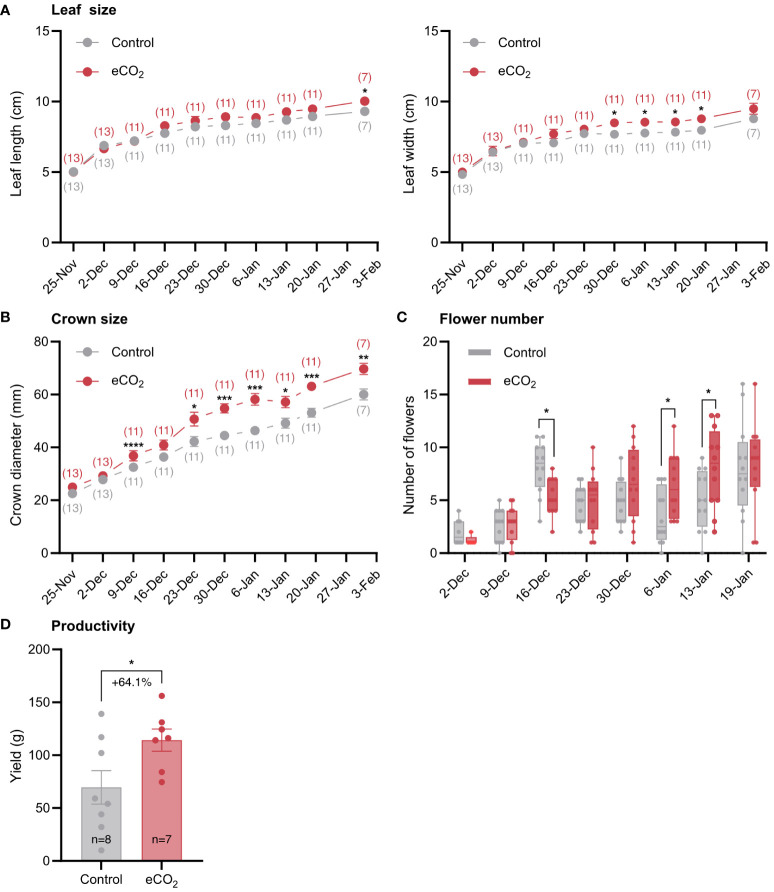
The effect of elevated CO_2_ on growth and yield-related parameters of strawberry plants. **(A)** Leaf length and width. The numbers in parentheses indicate the number of plants used in the analysis. **(B)** Crown diameter. **(C)** Number of flowers per plant. **(D)** Yield. n, number of plants used in the analysis. Error bars indicate the mean standard error, while dots in each bar represent the number of replicates. Percentage indicates changes in yield in response to CO_2_ enrichment. Significant differences (*P ≤* 0.05) between means were determined using t-test. The asterisks above data points indicate significant differences (*P ≤ 0.05, **P ≤ 0.01, ***P ≤ 0.001, ****P ≤ 0.0001).

Like tomato, eCO_2_ treatment promoted vegetative and generative growth of strawberry plants (**Figure 2**). Specifically, elevated CO_2_ levels resulted in increased leaf size as evidenced primarily by the increased leaf width and to a lesser extent the increased leaf length (**Figure 2A**). Elevated CO_2_ levels also increased crown diameter (**Figure 2B**), a trait that is positively correlated with yield, and the number of flowers per plant, especially after the second half of the flowering period (**Figure 2C**). The better performance of strawberry under elevated CO_2_ conditions (**Figures 2A–C**), was reflected on yield, which increased by 64.1% as a result of CO_2_ enrichment (**Figure 2D**).

### 3.2 Photosynthetic responses to elevated CO_2_

To determine the effect of elevated CO_2_ on photosynthetic responses of tomato and strawberry plants, we measured carbon assimilation rate (*A*), stomatal conductance (*g*_s_) and intercellular CO_2_ concentration (*c*_i_).

**Figure 3:**
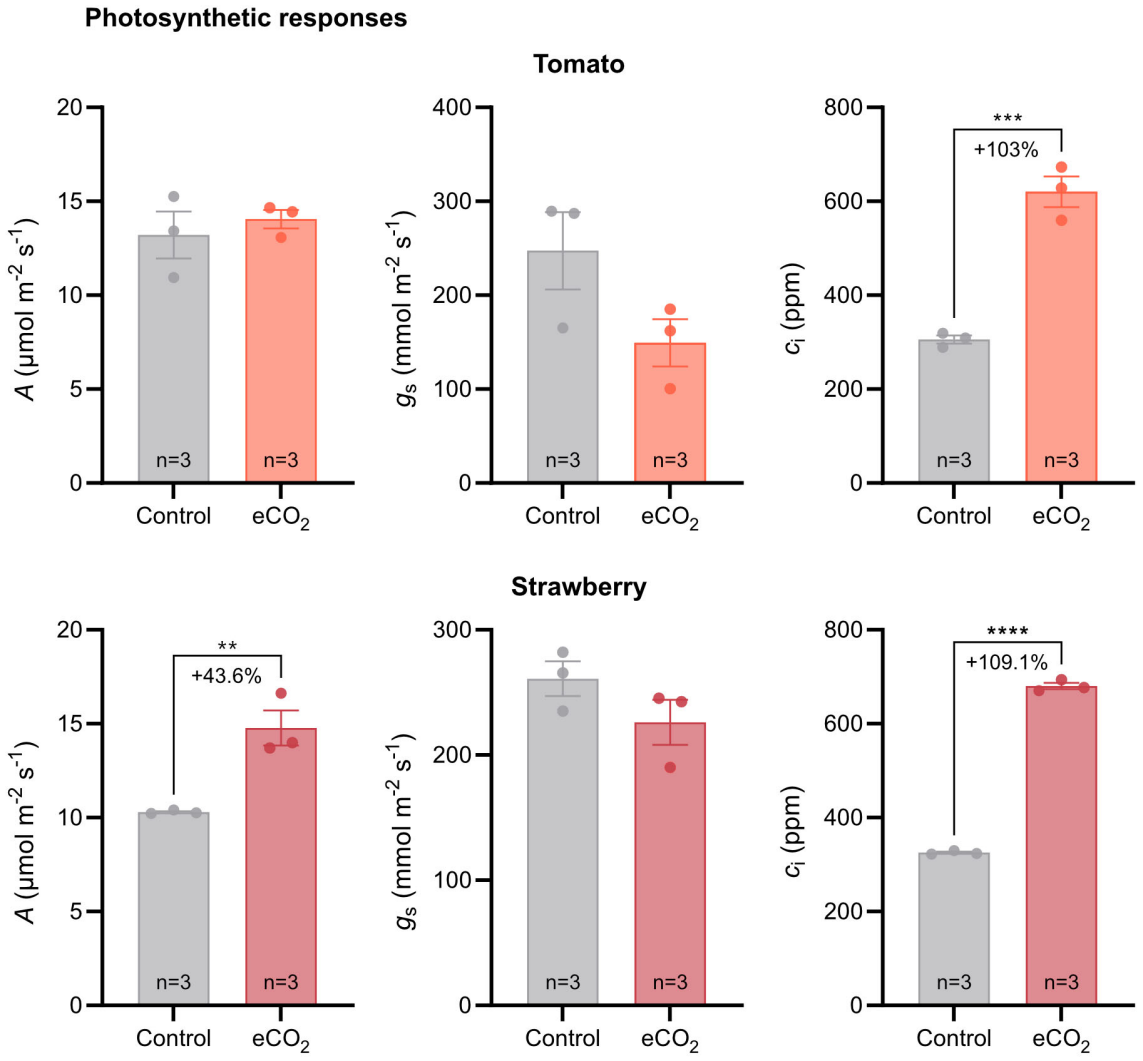
Photosynthetic responses of tomato and strawberry plants grown under ambient and elevated CO_2_ conditions. Error bars indicate the mean standard error, while dots in each bar represent the number of replicates (n). Percentage indicates changes in photosynthetic parameters in response to CO_2_ enrichment. Significant differences (*P ≤* 0.05) between means were determined using t-test. The asterisks above data points indicate significant differences (**P ≤ 0.01, ***P ≤ 0.001, ****P ≤ 0.0001). *A*, CO_2_ assimilation rate; *g*_s_, stomatal conductance; *c*_i_, internal CO_2_ concentration.

Tomato plants grown under elevated CO_2_ conditions had higher intercellular CO_2_ concentration in their leaves compared with plants grown under ambient CO_2_ conditions (**Figure 3**). The percentage increase in intercellular CO_2_ concentration in response to CO_2_ enrichment was 103%.Carbon assimilation rate and stomatal conductance, however, were similar between the two CO_2_ treatments (**Figure 3**).

In contrast, strawberry plants grown under elevated CO_2_ conditions had higher carbon assimilation rate and intercellular CO_2_ concentration compared with plants grown under ambient CO_2_ conditions (**Figure 3**). The percentage increase in carbon assimilation rate and intercellular CO_2_ concentration was 43.6% and 109%, respectively. No differences were observed in stomatal conductance between the two CO_2_ treatments (**Figure 3**).

**Figure 4:**
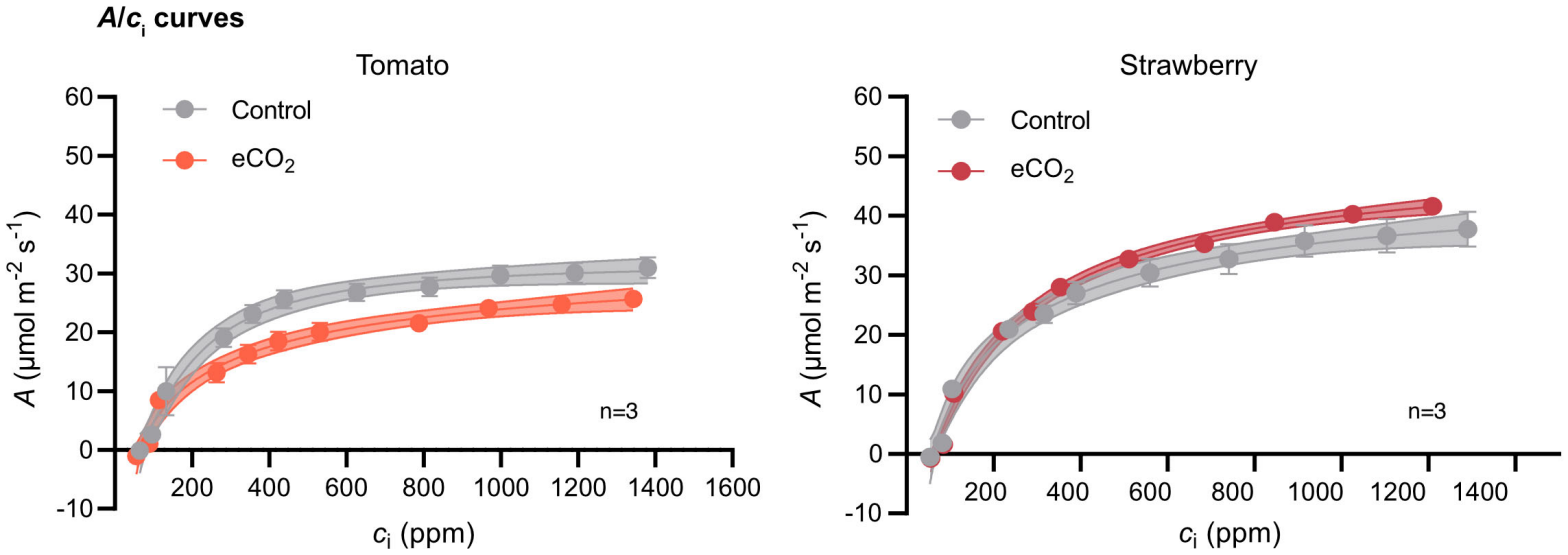
CO_2_ assimilation rate (*A*) as a function of internal CO_2_ concentration (*c*_i_) in tomato and strawberry plants grown under ambient and elevated CO_2_ conditions. *A*/*c*_i_ curves were generated using a photosynthetic photon flux density of (PPFD) of 1200 µmol m^-2^ s^-1^ and leaf temperature of 23°C. Each data point represents the mean value of three replicates ± SE, n=3. Dotted lines denote the 95% confidence interval.

To assess the extent to which Rubisco carboxylation efficiency influences carbon assimilation rate in tomato and strawberry, we estimated carbon assimilation rate as a function of CO_2_ concentration (*A*/*c*_i_ curves) at saturating light intensity (**Figure 4**). For both species, carbon assimilation rate increased with increasing CO_2_ levels until a certain concentration after which it reached a plateau. The CO_2_ concentration in which carbon assimilation rate saturated was lower in tomato compared to strawberry. Upon that, carbon assimilation rate was lower at elevated-CO_2_-grown tomato plants compared with ambient-CO_2_-grown plants at any given CO_2_ concentration, whereas the opposite trend was observed for strawberry plants (**Figure 4**).

### 3.3 Partitioning of newly assimilate carbon (^13^C) during fruit development under elevated CO_2_

Having established how elevated CO_2_ affects the phenotypic and photosynthetic responses in tomato and strawberry, we then labelled plants with ^13^C to understand how elevated CO_2_ influences the distribution of newly assimilated carbon to different organs.

**Figure 5:**
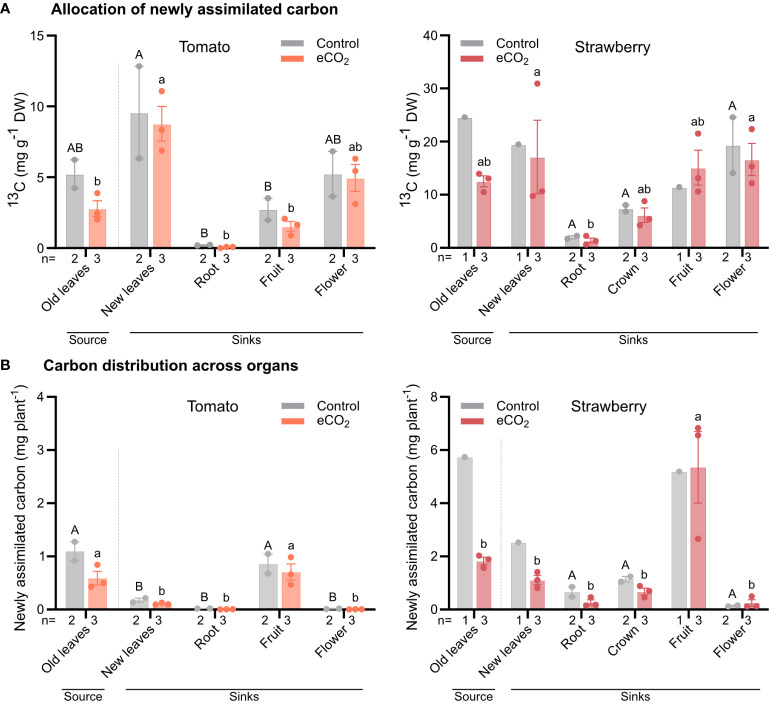
Newly assimilated carbon in individual organs of tomato and strawberry plants grown under ambient and elevated CO_2_ conditions. Plants were labelled for 2 h with ^13^CO_2_ and harvested after chasing for 24 h. ^13^C concentration was estimated in each individual organ **(A)**, and data were used to estimate the mean total ^13^C-assimilation in plants normalized to whole plant fresh weight **(B)**. Dashed lines separate source from sink tissues. Bars for control plants are the mean value of two replicates (n) ± SE, while bars for elevated CO_2_ plants are the mean value of three replicates (n) ± SE. Data were subjected to two-way ANOVA and significant differences (*P ≤* 0.05) between means were determined using Tukey’s test. Uppercase and lowercase letters denote differences between tissues from plants grown under ambient and elevated CO_2_ levels, respectively.

In tomato, under ambient CO_2_ conditions, all organs recovered a similar concentration of ^13^C, except for roots, which had the lowest ^13^C concentration (**Figure 5A**). Under elevated CO_2_ conditions, the highest concentration of ^13^C was found in developing (sink) leaves, followed by flowers, fruits and developed (source) leaves, and lastly roots (**Figure 5A**).

A similar allocation pattern to that found in tomato, was also observed in strawberry under ambient CO_2_ conditions (**Figure 5A**). However, under elevated CO_2_ conditions, all organs had a similar ^13^C concentration, except for roots, which again had the lowest concentration (**Figure 5A**).

When considering the mass of the different organs to estimate the relative distribution of newly assimilated carbon, we found that, in tomato, the majority of new assimilates remained associated with the developed (source) leaves or were allocated to fruits in both CO_2_ treatments, indicating that fruits are stronger sinks than other organs (**Figure 5B**).

Similarly, in strawberry, most new assimilates were allocated to fruits, regardless of CO_2_ treatment. For the remaining organs, new assimilates were equally distributed among them in both CO_2_ treatments (**Figure 5B**).

## 4 Discussion

One of the key findings of this study is that elevated CO_2_ treatment promoted growth and reproductive development in both tomato and strawberry. In tomato, the higher yield (**Figure 1E**) can be attributed to the higher number of inflorescences (**Figure 1B**), but not to the number of fruits per inflorescence (**Figure 1C**). In strawberry, the higher yield at elevated CO_2_ conditions (**Figure 2D**) can be attributed to increased crown growth (**Figure 2B**), which enabled the formation of more flowers (**Figure 2C**), and presumably the number of fruits per plant. The findings agree with those of other studies that found an increase in flower and fruit number in both tomato and strawberry under elevated CO_2_ (Deng and Woodward, 1998; Mamatha et al., 2014; Rangaswamy et al., 2021; Tagawa et al., 2022). Overall, these data suggest that elevated CO_2_ levels in the atmosphere will have a positive impact on the yield of field-grown tomato and strawberry crops in the future and support current glasshouse management practices entailing CO_2_ enrichment.

To determine the underlying mechanisms responsible for yield improvements under elevated CO_2_ conditions and to identify potential barriers limiting yield, we examined tomato and strawberry photosynthetic responses to ambient and elevated CO_2_ conditions. Interestingly, gas exchange analysis showed that carbon assimilation rate was higher in strawberry plants under elevated CO_2_, but there was no difference between tomato plants grown under ambient and elevated CO_2_ (**Figure 3**), despite the higher levels of CO_2_ in the mesophyll cells of elevated-CO_2_-treated tomato plants (**Figure 3**). The latter could be explained by the decrease in Rubisco’s carboxylation efficiency of tomato plants subjected to elevated CO_2_ compared with plants grown under ambient CO_2_, as evidenced by the lower CO_2_ assimilation rate of the former to increasing CO_2_ levels (**Figure 4**). In contrast, strawberry plants subjected to elevated CO_2_ conditions not only had higher CO_2_ concentration in the mesophyll space of their leaves, but also higher Rubisco carboxylation efficiency compared with plants grown under ambient CO_2_ conditions. The latter observation indicates that the symplastic loader, strawberry, can better adjust its photosynthesis to elevated CO_2_ levels than the apoplastic loader, tomato. Ainsworth and Lemonnier (2018) reported the results of a meta-analysis in which they compared the photosynthetic acclimation of apoplastic and symplastic species that had been studied to date in Free Air CO_2_ Enrichment (FACE) experiments. They found that the light-saturated photosynthetic rate, the maximum rate of Rubisco carboxylation (*V*_c, max_), and the maximum rate of electron transport (*J*_max_) of symplastic species were significantly higher than that of apoplastic species, and they attributed those differences to the better adaptation of passive loaders to high mesophyll sugar concentrations, which in turn renders them less susceptible to carbohydrate-mediated downregulation of photosynthesis. The findings of this study agree with those of Ainsworth and Lemonnier (2018).

In agreement with this study, Bishop et al. (2018) observed an increase in carbon assimilation rate in strawberry plants grown at elevated CO_2_ conditions; however, they also found an increase in carbon assimilation rate in all three apoplastic species as a result of elevated CO_2_ levels in the atmosphere. This contrasts with the findings of our work, in which we observed a similar carbon assimilation rate between tomato plants grown under either ambient or elevated CO_2_ conditions. One reason for that discrepancy might be associated with differences among apoplastic species in altering their photosynthetic responses to elevated CO_2_ levels. Another reason could be related to the fact that tomato plants in this study were only lightly pruned, resulting in higher shoot-to-root ratio and thus lower sink strength. This alteration in source-sink relationship in tomatoes may have disrupted the balance between production of sugars in source leaves and their utilization in sink organs. Indeed, as Arp (1991) indicated, whereas photosynthesis of C3 plants is stimulated when the CO_2_ levels in the atmosphere increase, their photosynthetic capacity is often reduced after long-term exposure to elevated CO_2_ levels, especially under conditions of low sink strength. The latter explanation is further supported by the data from ^13^C-labelling experiment in which we found that the distribution of newly assimilated carbon to sink organs was similar between tomato plants grown under ambient and elevated CO_2_ conditions (**Figure 5B**), suggesting that tomato plants are sink limited under elevated CO_2_. Hence, future breeding efforts, focusing on improving tomato productivity at elevated CO_2_ conditions, could revolve around enhancing sink strength, particularly that of flowers and fruits, and therefore the utilization of photoassimilates by harvestable organs.

Photosynthesis is generally considered a major determinant of yield, providing heterotrophic organs with carbon skeletons and energy for growth and development (Jones et al., 2013; Ort et al., 2015; Petridis et al., 2018). Here, we found that carbon assimilation rate was unaffected in tomato, but increased in strawberry, in response to long-term CO_2_ enrichment (**Figure 3**). Hence, based on their photosynthetic response, one would expect that tomato plants grown under elevated CO_2_ would have had similar yields to those grown under ambient CO_2_, whereas CO_2_ enriched strawberry plants would have had higher yields compared to those grown under ambient CO_2_. However, we found that not only strawberry plants, but also tomato plants had higher yields as a result of CO_2_ enrichment (**Figures 1**, **2**). In addition, we also observed that carbon distribution across organs was similar between treatments in both species (**Figure 5B**). If both species increase their yield in response to elevated CO_2_, irrespective of carbon assimilation rate and carbon distribution, then a question that arises is what triggers them. Although further research is needed to answer this question, we speculate that the yield benefits may arise from early developmental events, probably prior to photosynthetic acclimation to elevated CO_2_, promoting floral initiation and differentiation and thus the formation of more flowers. If this hypothesis is true, then it would be interesting to investigate the connection between photosynthesis and early stages of development, especially in relation to the maturation rate of shoot apical meristem, as this process can be a major driver of yield by altering inflorescence architecture and the number of flowers on inflorescences (Lippman et al., 2008; Park et al., 2012, 2014).

## 5 Conclusions

Our work confirms previous studies indicating that CO_2_ enrichment enhances strawberry and tomato yields (Mamatha et al., 2014; Tagawa et al., 2022). For both species this improvement in yield was the result of greater vegetative growth and flower formation. We also found that at elevated CO_2_ conditions carbon assimilation rate in tomato was limited by Rubisco’s carboxylation efficiency, but this limitation was not observed in strawberry. Furthermore, in both species most newly assimilated carbon had been allocated to fruits during fruit ripening; however, neither species allocated more carbon to sink organs of CO_2_-enriched plants, suggesting that the observed yield improvements in response to CO_2_ may have resulted from differences between treatments at earlier developmental stages. The similar response between strawberry and tomato plants, that is both species had similar levels of newly assimilated carbon in their sink organs between the two CO_2_ treatments, may additionally suggest that symplastic and apoplastic species are limited by their ability to export carbon at elevated CO_2_ conditions, at least at later developmental stages. Finally, based on this as well as previous studies (Bishop et al., 2018), we conclude that the phloem loading mechanism does not seem to affect crop adaptation to elevated CO_2_ per se, but any potential differences among species should be attributed to the individual ability of a species to adapt to elevated CO_2_.

## Data availability statement

The raw data supporting the conclusions of this article will be made available by the authors, without undue reservation.

## Author contributions

AP: Conceptualization, Funding acquisition, Investigation, Project administration, Resources, Supervision, Writing – original draft. MD: Data curation, Formal analysis, Investigation, Methodology, Writing – review & editing. SK: Data curation, Formal analysis, Investigation, Writing – review & editing.
